# Microbially Mediated Ore-Forming Processes and Cell Mineralization

**DOI:** 10.3389/fmicb.2019.02731

**Published:** 2019-12-03

**Authors:** Márta Polgári, Ildikó Gyollai, Krisztián Fintor, Henrietta Horváth, Elemér Pál-Molnár, João Carlos Biondi

**Affiliations:** ^1^Research Centre for Astronomy and Geosciences, IGGR, Budapest, Hungary; ^2^Department of Natural Geography and Geoinformatics, Eszterházy Károly University, Eger, Hungary; ^3^Department of Mineralogy, Geochemistry and Petrology, Szeged University, Szeged, Hungary; ^4^Polytechnic Center, Geology Department, Federal University of Paraná State, Curitiba, Brazil

**Keywords:** geomicrobiology, cell mineralization, EPS mineralization, ore-forming processes of Fe- and Mn, diagenesis

## Abstract

Sedimentary black shale-hosted manganese carbonate and oxide ores were studied by high-resolution *in situ* detailed optical and cathodoluminescence microscopy, Raman spectroscopy, and FTIR spectroscopy to determine microbial contribution in metallogenesis. This study of the Urucum Mn deposit in Brazil is included as a case study for microbially mediated ore-forming processes. The results were compared and interpreted in a comparative way, and the data were elaborated by a complex, structural hierarchical method. The first syngenetic products of microbial enzymatic oxidation were ferrihydrite and lepidocrocite on the Fe side, and vernadite, todorokite, birnessite, and manganite on the Mn side, formed under obligatory oxic (Mn) and suboxic (Fe) conditions and close to neutral pH. Fe- and Mn-oxidizing bacteria played a basic role in metallogenesis based on microtextural features, bioindicator minerals, and embedded variable organic matter. Trace element content is determined by source of elements and microbial activity. The present Urucum (Brazil), Datangpo (China), and Úrkút (Hungary) deposits are the result of complex diagenetic processes, which include the decomposition and mineralization of cell and extracellular polymeric substance (EPS) of Fe and Mn bacteria and cyanobacteria. Heterotrophic cell colonies activated randomly in the microbialite sediment after burial in suboxic neutral/alkaline conditions, forming Mn carbonates and variable cation-bearing oxides side by side with lithification and stabilization of minerals. Deposits of variable geological ages and geographical occurrences show strong similarities and indicate two-step microbial metallogenesis: a primary chemolithoautotrophic, and a diagenetic heterotrophic microbial cycle, influenced strongly by mineralization of cells and EPSs. These processes perform a basic role in controlling major and trace element distribution in sedimentary environments on a global level and place biogeochemical constraints on the element content of natural waters, precipitation of minerals, and water contaminants.

## Introduction

In the quest of understanding “*Biogeochemical Constraints on Water Contaminants*,” it is important to consider the role of microbial life (in particular, bacteria and fungi) in the geological context of mineralization and mobilization processes, because the mechanisms governing such activities often supersede purely inorganic reactions.

The scavenging properties of microbially precipitated/influenced Fe and/or Mn oxides/hydroxides and other components of (toxic) heavy metals, such as As, Cu, Co, Ni, and Ba, may highlight and influence accumulation pathways and, thus, reduced mobility and extraction from an aquifer.

Our experience in researching sedimentary Fe-Mn ores called our attention to the basic role of microbially mediated processes and the effect of cell and extracellular polymeric substance (EPS) diagenesis in determining the main and trace element composition of rocks.

### The Two-Step Microbial Ore Formation Model and Comparison of Case Studies

The role of microbial processes in the formation and transformation of rocks is a topic that is gaining attention among researchers. One example of this line of study is the research of Polgári et al. (2012a, b) into the biogenetic origin of manganese. Her work focuses on the principle of material-shape-process and is case study based. For instance, she has created a genetic model of the Úrkút site, which cast a new light on the formation of manganese ore in a black shale environment. This two-step microbial ore formation model has a chemolithoautotrophic cycle I under obligatory oxic conditions, which in the case of manganese precipitates metal ions from the aquatic system to solid form, and a heterotrophic cycle II, under suboxic conditions forming metal carbonates (rhodochrosite and siderite) under suboxic conditions (Polgári et al., 2012b). She also developed a new method for estimating the formation period based on a series of mineralized microbial population cycles. The basic principle of the method is that the microbial species mineralize only at a certain stage of the population growth periods (Polgári et al., 2012a).

This model was used successfully to detect the formation of iron ore in Rudabánya, Hungary (Bodor et al., 2016) and in the study of microbial Mn formation in the vicinity of ophiolites in the Abadeh-Tashk region of Iran (Rajabzadeh et al., 2017). The method was also used to estimate the time necessary for melting after the last glacial period in the Neoproterozoic Otavi Formation in Namibia, where climatic changes responsible for the melting were studied in the iron-biomat layers (Gyollai et al., 2015). The model was also used successfully in a Neoproterozoic Chinese black shale-hosted Mn carbonate deposit of the Datangpo area (Yu et al., 2019), and research is continuing on the Neoproterozoic Brazilian Urucum Mn and Fe oxide deposit (Banded Iron Formation). The Úrkút Model represents an important complex biomineralization methodology in international research. The question is whether it can be fully applied to certain similar sites.

**FIGURE 1:**
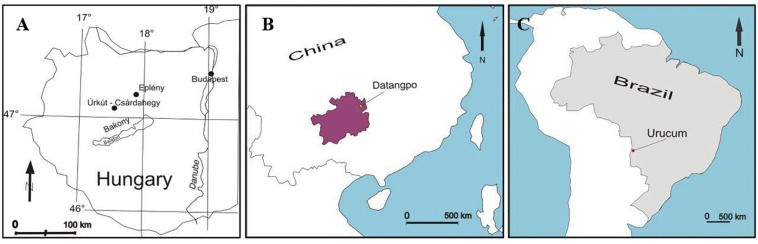
Localities of Mn deposits considered for comparison. **(A)** Úrkút, Hungary. **(B)** Datangpo, China. **(C)** Urucum, Brazil.

In this paper, we provide a comparison of results published in several case studies in three deposits including black shale-hosted Mn carbonate deposits of Neoproterozoic Datangpo, China (Yu et al., 2019), the Jurassic Úrkút manganese occurrences, Hungary (Polgári et al., 2012a, b, 2013, 2016a, 2016b, 2017), and the Neoproterozoic jaspilite and ironstone-hosted basically Mn oxide deposit, Urucum, Brazil (Biondi and Lopez, 2017; Biondi et al., 2019; Figure 1). The case studies are supplemented by a detailed high resolution study on the Brazilian Urucum Mn-1 bed, to characterize fine scale diagenetic element-mineral transformations supporting the important role of decomposition and complex mineralization of cell and EPS material.

Before going into depth on the recent case studies, a short overview on some aspects of biomineralization will help to shed light on these complex processes.

### Environments of Microbial Metal Accumulation

Microbes can play an intermediary role in ore formation (Ehrlich, 1999; Southam and Saunders, 2005). This role is especially important in low-temperature systems like sedimentary iron ore formation (Konhauser et al., 2002), in the wave front of uranium deposits (Nash et al., 1981), or in the formation of deep sea manganese nodules (Heath, 1981; Ehrlich, 2002). What environmental conditions support microorganisms in ore formation? What are the geo-environments where a significant amount of ore can be formed as a result of microbial activity? A short overview summarizes those environments, where metal accumulation via microbial mediation can be proposed.

#### Marine Environments

The largest number of microorganisms is known in aquatic systems (Fenchel et al., 2000). Research conducted in oceans has found important microbial activity in geological processes in deep sea sediments, gas hydrate deposits, as well as hydrothermal discharge vent systems (e.g., Dong and Yu, 2007). The highest level of microbial activity is typically found around the “geochemical hot spots” (black smokers, white smokers) (Parkes et al., 2000), and is caused by thermophile and hyperthermophile archea and bacteria (Miroshnichenko and Bonch-Osmolovskaya, 2006). Under these conditions, the microorganisms have to survive the pressure of a greater than 2500 m water column, the extremely high temperature of the emitted hydrothermal water (380°C), and toxic gases. The water of hot vents mixes with the colder, oxygen-rich sea water in the mixing zones, which helps microbes tolerate and survive these extreme conditions.

The smokers generally emit H_2_S-bearing gases, which represent one of the most important electron donors for the microorganisms living there. These localities are often covered by a centimeter-scale thick carpet of sulfur bacteria (Konhauser, 2007). The smokers discharge rich fluids bearing Fe (II) and Mn (II) and finely disseminated metal sulfides, so thick Fe- and Mn-oxihydroxides accumulate along these centers. Mineralization is partly the result of the activity of metal-oxidizing bacteria (Mandernack and Tebo, 1993).

A few meters distant from the smokers, there are also groups of organisms. The sulfide-rich smokers offer resources for tube worms, which are in symbiosis with sulfur-oxidizing bacteria, offering organic carbon for the bacteria, which in turn produce elemental sulfur, and Fe sulfide precipitation (Paradis et al., 1988). In methane-rich areas, giant bivalvia and methane-oxidizing bacteria live in symbiosis (Knoll et al., 2012).

The type of volcanic massive sulfide deposits related to hydrothermal processes originates via submarine volcanism, during which Cu, Zn, Pb, Ag, and Au enrichments form (Little et al., 2004). Such environments occur around submarine calderas related to volcanic–tectonic faults and in the fault zones of oceanic ridges. Bacterial sulfate reduction and Fe-oxidizing microorganisms also occur in these environments (Reysenbach and Shock, 2002; Edwards et al., 2004).

In the case of sedimentary exhalative deposits, laminated precipitation forms from the ore-forming hydrothermal fluids; the most common ores are Pb, Zn, Ag, Cu, and Au ores. Microbially mediated Zn-Pb-Ag deposits are also of economic importance (Schroll and Rantitsch, 2005). Cu deposits are known worldwide, which formed by similar bacterial activity, for example, the El Soldado deposit in Chile (Wilson et al., 2003).

#### Terrestrial Environments

An important ore-forming environment can form in the deeper parts of terrestrial areas (Dong and Yu, 2007). Anaerobic microorganisms favor these conditions, which are characterized by high or low pH, high pressure and temperature, radioactivity (radiation), and salt concentration (Rothschild and Mancinelli, 2001). The most common forms are anaerobic thermophilic and chemolithotrophic microorganisms (Dong and Yu, 2007). Dong and Yu (2007) distinguished three groups of these organisms based on their living conditions: (i) underground hot springs, (ii) oil reservoirs, and (iii) in metamorphic and magmatic rock habitats.

Important metal sources for microorganisms are the so-called red bed sediments (Kharaka et al., 1987; Warren, 2000): sandstones and claystones whose red color is caused by their Fe-oxide content. Biogenic H_2_S can increase the dissolution of metals and metalloids that bind to weak Lewis acids (Langmuir, 1997). Thus, microorganisms have an important role in ore formation by forming metal-rich ore-forming fluids (Southam and Saunders, 2005).

In geothermal fields, the sulfur-oxidizing metabolism is the most important one in the solfatara and hydrothermal discharge zones, because the reductive sulfur components are important electron donors for creating organic carbon components (Kletzin et al., 2004; Friedrich et al., 2005; Dong and Yu, 2007). These bacteria use geoenergy for their metabolism (Pedersen, 2001; Chapelle et al., 2002).

Extreme salty environments are also potential facies for microbially mediated ore-forming processes (Oren, 1999). The characteristic prokaryotic and eukaryotic organisms here are the halophiles (DasSarma and Arora, 2001), which have adapted to extremely salty conditions via evolution (heterotrophic and methanogenic archea, photosynthetic bacteria) (Dong and Yu, 2007). Archea are more frequent than bacteria, because they better tolerate high salt concentration (Ventosa et al., 1998).

In dry deserts, there are ore-forming microorganisms in spite of the change of hot and cold temperature and the low concentration of water (Wynn-Williams, 2000). The heterotrophic forms in these environments currently live under the surface at 25–30 cm depths (Drees et al., 2006) and take part in the long-lasting formation of sulfate deposits (Cooper and Mustard, 2002; Gendrin et al., 2005; Aubrey et al., 2006).

This study focuses on manganese and iron accumulation processes mediated by microbial activity, provides an overview on microbial activity and also the role of EPS, and gives a more comprehensive understanding of element distribution in geological substances. The study elaborates the main features of complex microbially mediated ore formation, discussing the effect of mineralization of cell and EPS material in the ore-forming conditions. Besides a comparison of manganese deposits of variable geological ages and types, we offer a new dataset from our recent investigation in the Brazilian Urucum deposit.

### Geomicrobiological Background

Geomicrobiology focuses on the study of microbiological processes that are part of and influenced by geological processes, and, as such, is one of those academic disciplines that combines the most up-to-date concepts. It is related to the scientific branches of geology and microbiology, and shows that microbial activity is either directly or indirectly involved in the transformation of organic and inorganic compounds, thereby facilitating the continuous circulation of elements in the biosphere (Southam and Saunders, 2005; Knoll et al., 2012). Thus, it is obvious that the activity of microorganisms affects the environment, and vice versa; certain environmental conditions affect microbial activity. This section discusses the differences among bacteria and the geomicrobiological significance of the communities they create.

Researchers typically apply the “theory of the 3 domains” (Woese et al., 1990), according to which cellular life can be divided into three large groups: *Archaea*, *Bacteria*, and *Eucarya*.

Organisms belonging to the *Archaea* and *Bacteria* domains are organisms that, unlike eukaryotes, do not have a nucleus with a real membrane, and as such, they are the simplest cellular organisms. Due to the lack of intracellular cellular organs, important metabolic and biosynthetic reactions take place at the periphery of the cell. Their size is generally in the micrometer size range (Stetter, 1999; Schulz and Jorgensen, 2001), they are unicellular, but the cells often cluster in different shapes. Nonetheless, the cells are not differentiated; they are protected by their own cell communities forming into micro-colonies (Donlan and Costerton, 2002). The *Archaea* and *Bacteria* domains together form the prokaryotes.

According to Schopf (1993), *Archaea* were already viable on Earth 3–4 billion years ago and geochemically influenced lithospheric, hydrospheric, and atmospheric processes. *Archaea* differ from real bacteria in their structure (Woese et al., 1990). Some species have mainly polysaccharides, proteins, and glycoproteins in the cell wall, but it can be clearly demonstrated that murein appears in different ways in the cells of the ancestral and true bacteria. In the case of true bacteria, unusual amino acids and N-acetylmuramic acid are found in the cell wall, which is called murein. In contrast, in the case of ancestral bacteria, no unusual amino acids are found, and the presence of N-acetyltalosaminuronic acid is typical. This is called pseudomurein. The cross-linked giant molecule defining the shape of the bacterium, the peptidoglycan, is absent from the cell wall of ancestral bacteria. The cell membrane of real bacteria consists of a double lipoprotein layer, which contains phospholipids and proteins, while the cell membrane of ancestral bacteria contains glycerol esters, and isoprene derivatives. In the case of the former (real bacteria), sterol is substituted with hopanoids while none of them can be found in the latter (ancestral bacteria). Their genetic material is also different because no introns have been found in the DNA chain of true bacteria, but introns can be found in ancestral bacteria, similarly to eukaryotic cells. In summary, the cell wall composition, cell membrane composition, genome organization, and ribosomal RNA sequences of ancestral bacteria differ from those of true bacteria. Ancestral bacteria are most likely to share a common ancestor with true bacteria, but they have evolved in a separate evolutionary way (Forterre and Philippe, 1999).

Eukaryotes are organisms whose cells have a nucleus. Already on the basis of this feature, they belong to a higher domain than the organisms of the previously discussed domains. Their cells are quite varied, ranging from 5 to 100 μm in size, and the cells are separated from the outside world by two different membranes. Their own DNA, ribosomes, and protein synthesis mechanisms make it possible for the cells to move independently of the nucleus. Their ribosomes and protein synthesis mechanisms resemble those of some prokaryotes; therefore, some scientists believe that prokaryotic cells once entered eukaryotic cells and lived endosymbiotically from that time on (Margulis, 1981). Based on these features, eukaryotes represent a different, more complex, alternative adaptation strategy, having a more differentiated intracellular structure. In the cytoplasm membrane of the cell, the isolated phosphate compounds are located on the outer surface of the glycerin, thus giving a negative charge to the cell. Metals are present either as positively charged ions in solution or in bound form as a solid component. In the metabolic processes of microorganisms, redoxidative reactions involving electron transfer take place, that is, the organisms can either oxidize or reduce certain metal ions (Southam and Saunders, 2005).

In geological environments, mineral formation not only can occur as an abiotic process, it can also be biotic as a result of the metabolic processes of bacteria (Macaskie et al., 1987, 1992; Rancourt et al., 2005). Abiotic processes are mineral depositions from metallic fluids. However, it should not be overlooked that, in nature, many secondary mineral formation processes take place more quickly and more efficiently than under laboratory conditions, as microorganisms are able to catalyze processes by 10^5^–10^6^ times (Lowenstamm, 1981). There are two possible locations for metal ion accumulation in cells: (i) bacterially controlled mineralization (BCM) (Bazylinski et al., 1988), which is bacterially controlled, direct mineralization within the cell (Pósfai et al., 1998) or (ii) bacterially induced mineralization (BIM) (Beveridge, 1989), which is bacterially induced, indirect mineralization outside the cell (Pósfai et al., 1998).

Among the differences between bacterially controlled and bacterially induced mineralization, it is important to note that within the cell, precipitation products have a maximum size of one or several nanometers. As a result, bacterial activity should not be attributed to a significant role in the formation of metal deposits (Southam and Saunders, 2005). In contrast, extracellular accumulation may be important in the formation of some ore deposits (Beveridge, 1989), as the organism can accelerate the accumulation of metals interacting with the bacterial surface by releasing reactive by-products into its environment (Tuttle et al., 1969; Fortin et al., 1994). The basic cases of extracellular mineralization are represented by the authigenic and the diagenetic methods of mineral formation (Ehrlich, 2002). In authigenic mineral formation, when a given mineral component reaches a certain concentration, it precipitates. In the course of diagenetic mineral formation, the reduction of materials and their reaction with cations may result in the formation of a substance that can be economically extractable in both quantity and concentration. In geological surveys, it is usually not possible to separate direct and indirect mineralization.

### Gel Matrix

Extracellular polymeric substances are highly hydrated structures, produced within the cell and excreted as gel or liquid substances on the cell surface (Konhauser, 2007). In fact, this material is called a gel matrix. Flemming and Wingender published an article in 2010 about what they really are and their role in the life of microorganisms. The topic is presented here based on their work.

In order to survive in the long term and sustain favorable living conditions, microorganisms usually do not appear as single cells in a given environment; rather, they accumulate on surfaces as polymeric aggregates. The different species create useful interactions based on each other’s life activities. For the long-term sustainability of these interactions, the colonies produce a hydrated matrix (biofilm).

The biofilm contains a total of 10% of microorganisms, with the remaining 90% being the matrix itself (Flemming and Wingender, 2010). In fact, EPS is the skeleton of this matrix, and it can be defined as a combination of polysaccharides, proteins, nucleic acids, and lipids (Flemming and Wingender, 2010). First of all, they facilitate adhesion to the surface and provide stability to the biofilm, but they also have numerous other functions, some of which are as follows: cell aggregation, cohesion, water retention, barrier function, nutrition source through the sorption of organic and inorganic ingredients, and excess energy absorption.

Biofilm-forming colonies may also appear in low-carbon-content deposits, in both ocean and freshwater conditions. Their structure can be varied because their formation depends on the species of the microorganisms forming them as well as their life activity, and on the response of the organisms to the environmental properties (such as hydrodynamics and nutrient concentrations). The morphology of the biofilm may be smooth, cloudy, flat, fluffy, or even filamentous, and even their degree of porosity may change. Despite the morphological diversity, the essence of each structure is to temporarily immobilize cells and to create a long-term existence of mixed-species micro-cultures. With EPS, biofilm can become one of the most successful lifestyles of the Earth, other than the planktonian way of life (Flemming and Wingender, 2010).

The cells of the microorganisms and the biofilm (EPS) they produce are able to interact with mineral surfaces, and after their decay, all their components are recycled and incorporated into biominerals. The presence of microorganisms in geological aggregates is generally difficult to determine, since the original materials transform, mobilize, and mineralize as a result of diagenesis. One feature to look for is microlamination, in which biominerals actually form a thin layer on the surface of the fossil EPS laminates, allowing us to assume microbial activity or the presence of fossil EPS in rocks. Autochton and allochton minerals can also be found in thin layers or between the layers.

Materials appearing in the original ancient environment may appear in the rocks to be examined in highly diverse ways as a result of diagenesis. The reason for this is that the EPS types present depend on the composition of the colonies, which basically determines the nature of the formed materials. During the analyses, we have the possibility of examining the diagenetic, that is, the transformed materials, and we have to deduce the processes from them in order to figure out the original materials. Each of the many diagenetic minerals found in the samples may have significance as an environmental indicator.

Through its functions mentioned above, the EPS gel matrix is most capable of capturing elements that can participate in the biological element cycle. It can bind metals and metal cations, on the basis of the ligand types of the cells (oxygen types can bind K^+^, Mg^2+^, Ca^2+^, and Fe^3+^ ions and their substitutes; sulfur- and nitrogen-type ligands can bind Ca^2+^, Zn^2+^, Cd^2+^, and Fe^2+^ ions and their substitutes). They can be bound not only through the metabolic processes of the microorganisms, but EPS, as a reactive surface, can enhance the precipitation kinetics of supersaturated solutions. The role of microorganisms is rarely considered to be significant in mineral or ore formation, as the biofilm they produce is only a millimeter thick (Ehrlich, 2002). However, it should not be forgotten that the biofilm can cover large surfaces and thus dominate when the abiotic processes occur (Flemming and Wingender, 2010). In fact, they facilitate the sedimentation of metals by their adsorption from the water (Konhauser, 2007). Increasing biomat condensation and 3D mats are related to sediment starvation in the sense of debris contribution (Polgári et al., 2012b). The biomat system transforms to cyclic mineralized microbially produced textures and lamination via diagenesis.

After the death of the cells, the decomposition of cells and EPS starts and ions that are bound on their surface will liberalize. This begins a complex transforming mineralization, which can result in clay mineral formation, mixed carbonates, feldspar, pyroxene, hornblende, silica, apatite, sulfides, sulfates, etc., depending on the geochemical conditions. These poorly crystallized minerals can form more stable minerals in time (Biondi et al., 2019).

In Fe systems, metal adsorption on EPS results in the nucleation of small metal-hydroxide particles, and, after sufficient time, the cells are fully embedded and their organic residues are combined with mineral precipitations. Mineralization on the surface of dead cells may occur independently of the physiological state of the cell. When the primary metal-hydroxides precipitate and enter the sediment, a number of diagenetic reactions can modify their reactivity, morphology, and mineralogical properties. Dehydration and internal rearrangements as well as dissolution and precipitation processes result in more stable phases such as hematite, goethite or magnetite. Silica segregation accompanies the stabilization of ferrihydrite. Since microbial surfaces can also develop in a similar manner, minerals may be embedded in them (Knoll et al., 2012).

In Mn-rich systems, we need to consider abiotic processes that result in large deposits. However, in some cases, such as hydrothermal Mn colonies, it is generally accepted that the highest degree of Mn(II) oxidation occurs through microbial catalysis. In these environments, todorokite and birnessite are actively accumulated due to the flow of metal-rich fluids. Mn(II) oxidation by microorganisms living here can be linked to the energy intake of colonies (Konhauser, 2007).

Microorganisms can also contribute to the formation of clay mineral phases. The iron adsorbed on EPS serves as a kinetically beneficial nucleus for more complex and diverse clay mineral structures (negatively charged ions accumulate on the positively charged metal grid to neutralize it). Well-known inorganic processes naturally occur together with them, since aluminum and silica-saturated solutions precipitate weakly crystallized aluminum silicates into which iron can easily be incorporated (Konhauser, 2007). Al is offered by organic matter base, as reported by Maliva et al. (1999), showing that the aluminum content is greatly increased by complexing with organic acids.

Biofilms have been found in lots of geysers, where the layers were silicon dioxide, but it is well known that this process also occurs as an abiogenic process. The biogenic silification of such thermal springs occurs because the microorganisms grow in an apolymerizating medium (biofilm) where silification is inevitable (due to hydrogen bonds, cation bridge bonds, and electrostatic interactions) (Knoll et al., 2012).

Microorganisms can play an important role in the formation of carbonate sediments. Cyanobacteria often come into contact with calcite, aragonite, or dolomite. Their metabolic processes bind the inorganic carbon, which increases the pH of the solution so that the cations can bind to the cell surface in a solution that is oversaturated with carbonate that forms relatively easily. EPS can be an important biological surface that nucleates authigenic calcite, or traps authigenic granules as a protective net. In addition to anoxic, hypersaline conditions, sulfate-reducing agents are able to promote dolomite formation, as the reduction of sulfate increases Mg^2+^ levels and dolomite precipitates around the micro-environment. Microorganisms may also be responsible for precipitation of siderite and rhodochrosite, and may even result in the formation of numerous authigenic minerals by altering the Eh and pH conditions of their environment (Ehrlich, 2002).

Cell conservation is provided by the minerals around the cells and EPS. In a geological sample, most microorganisms are well preserved in cryptocrystallized quartz material, because this small mineral can cover the cell and protect it during the process of diagenesis. The role of EPS in cell fossilization is similar, as it also provides a protective function, and can even preserve cells by its peptidoglycans and polymers (these compounds can withstand degradation, which may explain the metastable state of these minerals) (Konhauser, 2007).

In many cases, biogenic minerals may appear to be the same in appearance as abiogenic minerals, which makes it difficult to understand the conditions under which minerals were formed. The reason for this is that the processes operate on the basis of the same thermodynamic principles. In order to make well-founded statements about the biogenic origin, we need a comprehensive, complex, high-resolution study, and system-based approach (Knoll et al., 2012).

The verification of biogenicity in terrestrial systems is a very important question; recent analogies are also being used for interpretation of geological samples. But the interpretation of biosignature on a geological scale (Ga-My) is not easy, since diagenesis and other processes can overprint microbial features. The following features are proposed as indicators of microbial origin in geological samples, which require multi-methodological high-resolution investigation and complex interpretation (Cady et al., 2003):

(1) Microbial microtexture: filamentous, coccoid like, vermiform, brain-like, and stromatolite-like macrotexture – fine lamination multiple cyclicity.
(2) Bioindicator minerals that can be modified by diagenetic and other processes.
(3) Presence of organic matter embedded in minerals.
(4) Biosignatures like isotope signals (“vital effect”) (C, S, N, and Fe, etc.), shape of minerals, and selective enrichment of bioessential elements (Fe, Mn Zn, As, Be, U, and P, etc.).
(5) Recent analogies of biomineralization, biomarker organic matter.
(6) Paleoenvironmental analogies (sedimentary, etc.).
(7) Preservation.

The following methods are suggested to study the abovementioned biosignatures:

(1) High-resolution polarization microscopy by adequate magnification of 40×, 100×, 200×, 400×, and 1000× for identification of both textural and micromorphological features of biomineralization.
(2) Spectroscopical methods for *in situ* determination of biominerals and organic material (Raman spectroscopy, ATR-FTIR spectroscopy, and gas chromatograph–mass spectrometry).
(3) Determination of isotope signals (C, S, and Fe) by mass spectrometry.
(4) Complex interpretation of data in a structural hierarchical system.

## Materials and Methods

### Sampling Locality of Brazilian Samples

The sampling locality is in southern Brazil, in the state of Mato Grosso do Sul, close to the Bolivian border, in the Urucum Mining District, where the Fe and Mn ores are located at 1000 m above sea level (detailed geological maps and sections are published by Biondi and Lopez, 2017). According to estimations, the ore reserve is approximately 600 million tons with an average of 27–44% Mn ore and 12–30% Fe ore (Urban et al., 1992).

#### Geological Setting

The geological setting mapped by Biondi and Lopez (2017) is the following: The manganese ore deposits are located in the Santa Cruz Formation. These sediments are rich in iron and manganese elements and fill a previous depression, which presently form a plateau. The Urucum Formation, the footwall to the Santa Cruz Formation, is a fluvial sediment, and together, these two formations create the Jacadigo Group. The limestone of the Corumba Group borders the Jacadigo Group, whose sediments were built up from the Bocaina and Tamengo Formations. The basement of these formations is granite and granodiorite, which contain hornblende and biotite. The granite suffered chloritization and sericitization along faults, and its color turned green. This change is probably the result of hydrothermal activity (Freitas et al., 2011).

The Urucum Formation, representing the alluvial series, is a 10-m-thick sandstone cemented by feldspar and interbedded with shale and dolomite conglomerate. This formation contains Mn carbonate cement and occasionally stromatolite structures. Above the Urucum Formation is located the Santa Cruz Formation, which includes jasperic iron ore, ferruginous arkose, and sandstone layers. The thickness of the ore layers varies from 40 m to 396 m in the sediment. The massive manganese ore deposited among these layers are named Mn-1, Mn-2, and Mn-3. The investigated samples of the Mn-1 ore bed contain quartz, feldspar, apatite, chert, and hematite clasts with manganese oxides. Both the Mn-2 and Mn-3 ore beds include centimeter- or decimeter-sized concentric structures of manganese oxides called kremydilites (Biondi and Lopez, 2017).

The Bocaina Formation is composed of limestone and dolomite with conglomerate layers. The Tamengo Formation consists of limestone, dolomite, shale, and aleurite. In this sediment, lens-like conglomerate occurs with carbonate and granite gravels. In the Bocaina Formation, stromatolites can be found. The Tamengo Formation includes specimens of Ediacara fauna (Boggiani et al., 2010). Pleistocene sediments are composed of limestone, travertine, sandstone, and conglomerate (Biondi and Lopez, 2017).

### Samples

**FIGURE 2:**
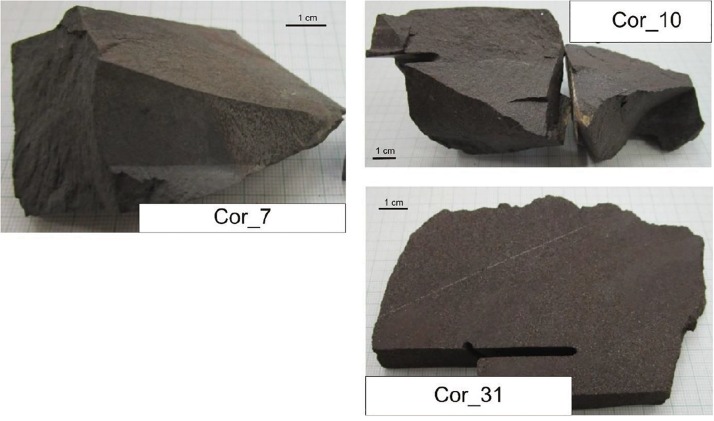
Representative samples from Mn-1 ore bed of Urucum Mn deposit. Samples COR-7 and -10 were collected at Figueirinha Mine, Santa Cruz N plateau, and sample COR-31 was collected at Saõ Domingos Mine, Santa Cruz SW plateau.

The representative samples and the methods used (number of photos and spectra) are summarized in Table 1 and Figure 2. Localities of the samples are Figueirinha Mine, Santa Cruz N plateau, and Saõ Domingos Mine, Santa Cruz SW plateau. The representative samples were collected from the Mn-1 ore bed by one of the authors, Joaõ Carlos Biondi, for investigation to clarify the complex mineralization of the ore in more detail (four samples). This investigation is a continuation of Biondi et al. (2019).

**TABLE 1:** Samples and methods used.

**Sample ID**	**Layer**	**Locality**	**TS^∗^**	**OM**	**CL**	**Raman**
COR-7	Mn-1	Figueirinha Mine, Santa Cruz N plateau	x	x(21)	x(12)	x(34)
COR-10 (2 subsamples)	Mn-1	Figueirinha Mine, Santa Cruz N plateau	x	x(17)	x(43)	x(191)
COR-31	Mn-1	Saõ Domingos Mine, Santa Cruz SW plateau	x(2)	x(7)	x(15)	x(37)
Total 4 samples (photos and spectra)				45	70	262

*^∗^TS, thin section; OM, optical rock microscopy; CL, cathodoluminescence microscopy; Raman, Raman spectroscopy. Number of analyses in parentheses.*

The samples of Mn-1 are COR-7, a very fine-grained clast-bearing ore with braunite and carbonate; COR-10 (two subsamples), a sandy, detritic ore with braunite, quartz, and feldspar; and COR-31, an arkosic sandstone with hematite matrix.

### Methods

Petrographic structural–textural studies by optical rock microscopy (OM) were made on four thin sections in transmitted and reflected light (NIKON ECLIPSE 600 rock microscope, Institute for Geology and Geochemistry, Research Centre for Astronomy and Earth Sciences, Hungarian Academy of Sciences – IGGR RCAES HAS, Budapest, Hungary). Forty-five photos and panoramic photo series were taken of all sections.

Cathodoluminescence (CL) petrography was carried out on the four thin sections using a Reliotron cold cathode CL apparatus mounted on a BX-43 Olympus polarization microscope (Szeged University, Hungary). Accelerating voltage was 7–7.7 keV during the analysis. CL spectra were recorded using an Ocean Optics USB2000 + VIS-NIR spectrometer. Spectrometer specifications are 350–1000 nm wavelength range and 1.5 nm (FWHM) optical resolution. Seventy photos were taken.

High-resolution *in situ* micro-Raman spectroscopy was used for micro-mineralogy and organic matter identification and distribution on the four thin sections, resulting in 262 spectra (Szeged University, Hungary). A Thermo Scientific DXR Raman Microscope was used, with a 532-nm (green) diode pumped solid-state (DPSS) Nd-YAG laser using 1.5-mW laser power, and a 50× objective lens in confocal mode (confocal aperture 25 μm slit). Acquisition time was 1 min and spectral resolution was ∼2 cm^–1^ at each measurement; the distance between each point was 10 μm and the measurement time was 10 min. A composite image of thin sections of Raman microscopy measurements is indicated on thin section photos (points and section measurements). Diagrams were organized on peak height versus analytical spot number of each of the phases along the Raman scanned section. Intensities were normalized to the highest peak for each spectrum.

Raman measurements were taken on four samples, four line profiles (lines 1, 2, 3, and 4 in Table 2) and random point analyses were acquired (Table 2). The spectra were elaborated in two ways: (1) Diagrams were organized on peak height versus analytical spot number of each of the phases along the Raman scanned section (main minerals and organic matter in general). The results of (1) are diagrams. (2) A detailed determination of all spectra was also carried out. The results of this are summarized in Table 2, in which we can follow the mineral composition and also the type of organic matter. Besides section analyses, mineral phase transitions were also made for clarification.

**TABLE 2:** Raman vibration of minerals and detected types of organic matter.

**Sample ID**	**Bands (Raman shift cm^–1^)**	**COR-7**	**COR-10**					**COR-31**	**References**
**No. of spectra (total: 262)**		**Random anal**	**Line 1**	**Line 2**	**Line 3**	**Line 4**	**Random anal**	**Random anal**	
		**34**	**16**	**41**	**41**	**39**	**54**	**37**	
**Fe minerals**									
Hematite	222, 290, 408, 490, and 607 hem	0	10	15	16	15	2	21	Das and Hendry, 2011
Goethite	162, 243, 297 s, 385 s, 477, and 545	0	2	0	3	0	0	15	Das and Hendry, 2011
Aegirine	185, 212, 341, 365, 387, 541, 661, 970 s, and 1040	0	0	4	20	7	0	0	RRUFF
Celadonite	192, 260, 380 m, and 545 s	0	0	0	0	0	1	0	Madejova and Komadel, 2001
Mn minerals									
Birnessite	246 m, 303, 506, 575, 656 s, 730, and 912 m	0	0	0	1	0	0	0	Julien et al., 2004
Ramsdellite	515, 650, and 756 m	0	1	0	0	0	0	0	RRUFF
Pyrolusite	219 w, 291 w, 404 w, 533 s, 655 s, and 756 w	0	1	0	0	0	0	0	Sepúlveda et al., 2015
Hausmannite	306, 374, and 661 s	0	0	0	2	0	0	0	RRUFF
Manjiorite	641 s	0	0	0	0	0	1	0	RRUFF
Jacobsite	620 s	0	0	2	0	0	0	0	RRUFF
Serandite	666 s, 700, 968, and 1015	0	0	8	0	0	3	0	RRUFF
Braunite	210 s, 331, 376 w, 510 m 622, 685, and 970	0	3	0	1	12	3	12	RRUFF
Rodochrosite	181, 287, 721, and 1087	2	0	1	0	0	0	0	RRUFF
Other									
Apatite	427, 587, 605 w, 965 s, 1040 w, and 1078 w	3	0	1	0	2		0	RRUFF
Feldspar (albite)	478 s, 507 s, 287 m, 330, 244 w, 207 sh, 182 m, and 161 sh	9	28	20	5	13	25	0	RRUFF
Feldspar (microcline)	267, 281, 455 m, 471 s, 514 s, 749, and 811	25	0	0	0	0	10	0	RRUFF
Quartz	125,207, 353, 393 w, and 464 s	0	17	10	14	8	12	21	RRUFF
Mica	259s, 400, and 703	0	0	0	0	0	3	9	RRUFF
Kaolinite	243, 268, 331, 422, and 452	0	0	0	0	0	0	4	Madejova and Komadel, 2001
Barite	446 and 985	0	7	12	0	0	4	0	RRUFF
Johannite	785 s	1	0	0	0	0	0	0	Frost et al., 2005
**Organic matter^∗^**									
Org1	824, 1069, and 1310	0	8	5	3	0	0	1	
	*Vibration types*								
	aromatic CH, CH in plane bending, and CH aliphatic band								
Org2	820, 1459, 1317, and 1600	0	0	0	0	13	0	1	
	*Vibration types*								
	Aromatic CH, CH2/CH3 bending, CH aliphatic band, and fluorene (aromatic CH)								
Org3	1162, 1380, and 1607	0	3	0	0	0	0	0	
	*Vibration types*								
	C = C breathing, CH3, and fluorene (aromatic CH)								
Org4	804 and 1162	0	5	15	6	0	16	0	
	*Vibration types*								
	aromatic CH, C = C breathing								
Org5	825, 1109, 1186, 1386, 1469, 1580, and 1607 *Vibration types* aromatic CH, aliphatic CH, C = C breathing, CH3, CH2/CH3 vibrational mode, quadrant CH stretching modes of cyclic aromatic ring system, fluorene (aromatic CH)	29	0	0	0	0	13	4	
Org6	804, 1107, and 1608	0	0	0	0	0	4	0	
	*Vibration types*								
	aromatic CH bend, aliphatic skeletal C-C, CH, fluorene (aromatic CH)								
Org7	682 m, 1146 s, 1054 s, 1361, 1395, 1480, 1527, 1717, and 1773	3	0	0	0	0		0	
	*Vibration types*								
	C = C ring modes, C = C breathing mode, C-H in plane vibration, CH3 modes, CH2/CH3 modes, C = C stretching in polyene chain, and C = O in oils								

*^∗^References: Orange et al. (1996), Jehlička et al. (2009), and Okolo et al. (2015). RRUFF, database of Raman spectroscopy, X-ray diffraction, and chemistry of minerals: http://rruff.info/.*

Identification of minerals was made by the RRUFF Database (Database of Raman spectroscopy, X-ray diffraction, and chemistry of minerals)^1^. Contamination by epoxy glue was taken into consideration.

Though, in the recent study of the Urucum deposit, AT-FTIR spectroscopy was not used, references include datasets, which were used in interpretation.

Comparing the two sensitive *in situ* methods (Raman and FTIR), it was AT-FTIR that did not modify considerably the mineral phases, using the lowest exciting energy when investigating the upper 1–2 μm of the samples. Also, this is the best method to determine organic matter. On the contrary, Raman spectroscopy with its higher excitation energy often caused transformation of metastable minerals to more stable phases. This method is getting information from the upper 3–4 μm depth of the sample surfaces and is the best one for identification of Mn oxide hydroxides. The comparative spectra database for Raman is larger than that for AT-FTIR. As a summary, the Raman spectroscopy and AT-FTIR are powerful methods to identify *in situ* bioindicator minerals and organic material as biomarkers of microbial structures. The advantage of the AT-FTIR method is the lower excitation energy, which makes it possible to determine poorly crystallized syngenetic microbially produced minerals like ferrihydrite and vernadite/hollandite.

In general, it is important to choose relevant methods that can detect poorly crystallized minerals without any transformation affect. XRD, for example, is a routinely used method for geological samples, but its usage in the case of X-ray amorphous material, like most microbially mediated samples, is not relevant.

## Results

### Optical Rock Microscopy

**FIGURE 3:**
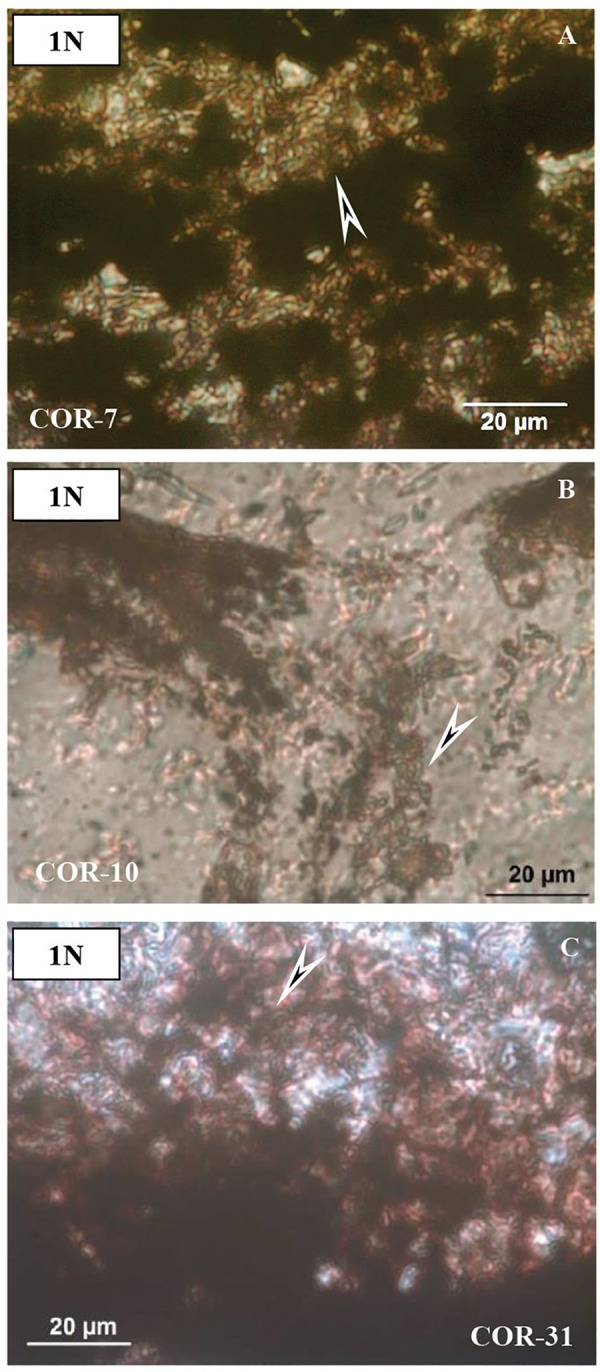
Microtextural features of samples by optical rock microscopy (OM). Mineralized microbial biosignatures (arrows). **(A)** Sample COR-7. **(B)** Sample COR-10. **(C)** sample COR-31 (transmitted light, 1 N).

Thin sections represent mineralized biomats based on structural observations. In all thin sections, adequately high-resolution OM (1000×) supports a series of mineralized biomat microstructures, with mineralized microbially produced textures (MMPT) as their main constituents (Figure 3). This microbial microtexture is a basic feature of all the samples, in transmitted as well as reflective light. Well-preserved and mineralized remains of diverse filaments with pearl necklace-like, vermiform inner signatures, and coccoid-like forms embedded in the Mn ore bed were observed, and all samples are densely woven. The minerals are very fine-grained (0.5–1 μm). The diameter of the mineralized filaments is around 0.5–1 μm, with variable length (Figure 3).

Samples 7, 10, and 31 include debris-like components of variable size (20–200 μm). The debris grains are mainly quartz with few fragments of jasper and altered feldspar.

**FIGURE 4:**
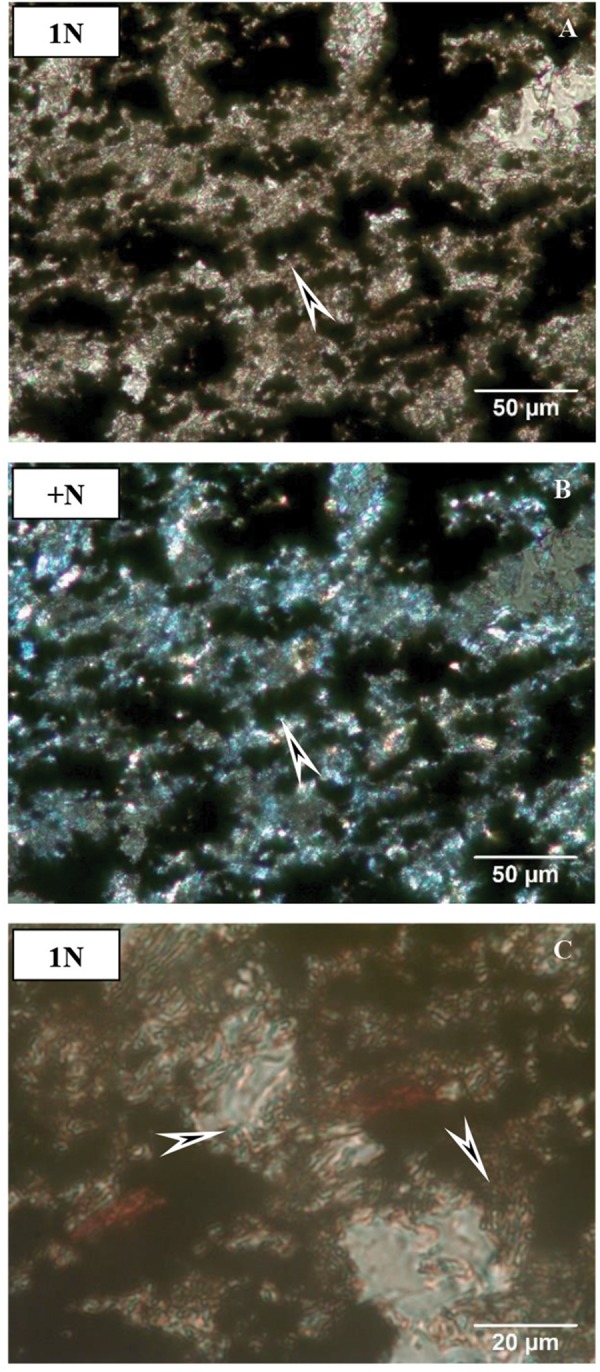
Microtextural features of sample COR-7 by OM. **(A)** Biomat-like forms (arrow, 1N, and transmitted light). **(B)** Crossed N of (A). **(C)** Mineralized microbial biosignatures (arrows, transmitted light, and 1N).

Sample COR-7 contains opaque grains (30%) that show dense microlamination (Figure 4). Along the microlaminae, we observed more small grains of maximum 50 μm in diameter, which represent 60% of the section. The remaining 10% is shapeless very fine-grained matrix material, and all of the other constituents are embedded in this matrix.

**FIGURE 5:**
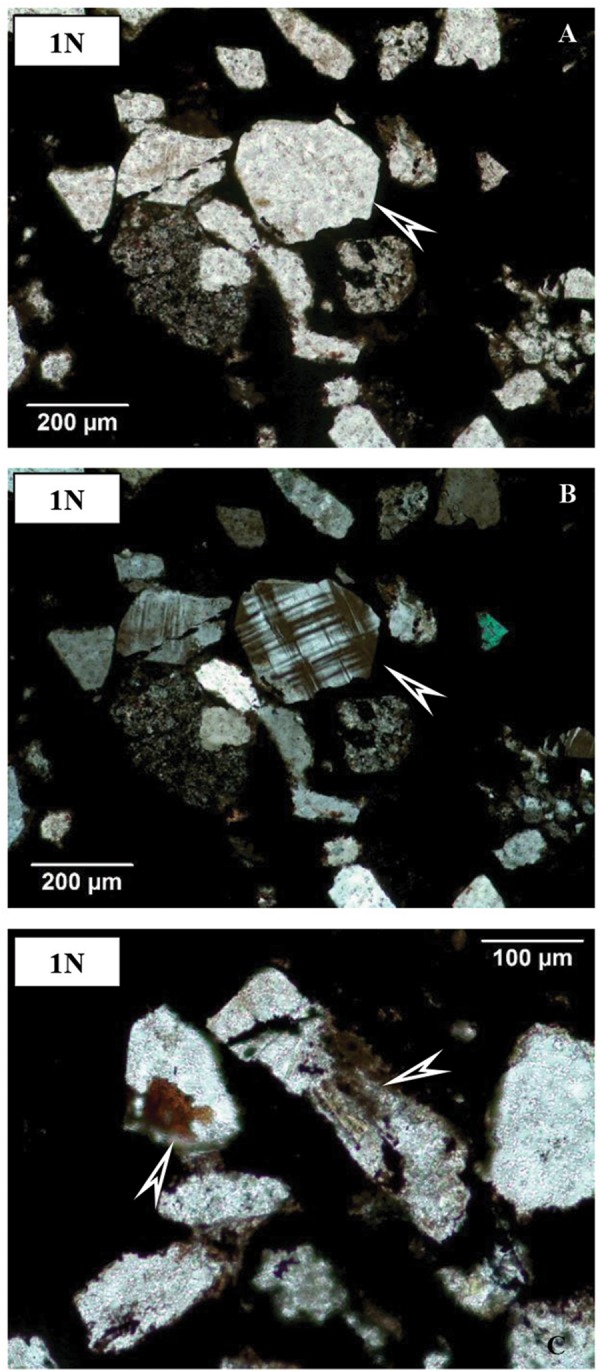
Microtextural features of sample COR-10 by OM. **(A)** Debris-like grains (mainly quartz, altered feldspar, and fragments of jasper) in opaque matrix (arrow, 1N, and transmitted light). **(B)** Crossed N of **(A)**, showing polysynthetic twin microtextures of plagioclase (arrow). **(C)** Brown mineralized microbial biosignatures along and inside debris-like grains (arrows, transmitted light, and 1N).

Sample COR-10 contains much more opaque material, estimated up to 70%, which is dense matrix material. In this matrix, smaller–larger grains occur up to 300 μm (30%). The shape of the grains is irregular and their margin is sharp or rounded. The surface of the grains is often covered by brown or green fine-grained material, and the grains become obscure. Polysynthetic twinning of plagioclase was observed in this sample (Figure 5).

**FIGURE 6:**
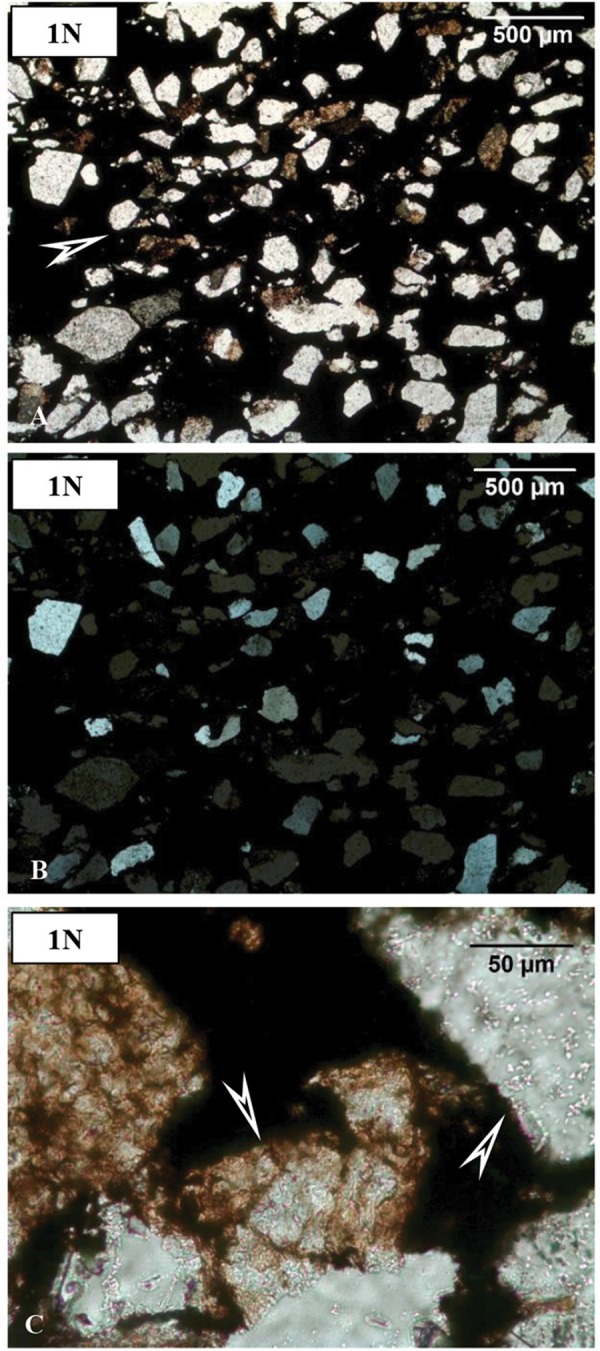
Microtextural features of sample COR-31 by OM. **(A)** Debris-like grains (mainly quartz, altered feldspar, and fragments of jasper) in opaque matrix (arrow, 1N, and transmitted light). **(B)** Crossed N of **(A)**. **(C)** Brown mineralized microbial biosignatures along and inside debris-like grains (arrows, transmitted light, and 1N).

Sample COR-31 resembles sample COR-10 rather than sample COR-7. The difference is the larger size of grains here, with grains up to 400–500 μm in diameter (Figure 6). The opaque matrix material present is estimated up to 50% of this sample. The shape of the grains is irregular and their margins are sharp and rounded. The debris-like grains include dense elongated inclusions.

### CL Rock Microscopy

**FIGURE 7:**
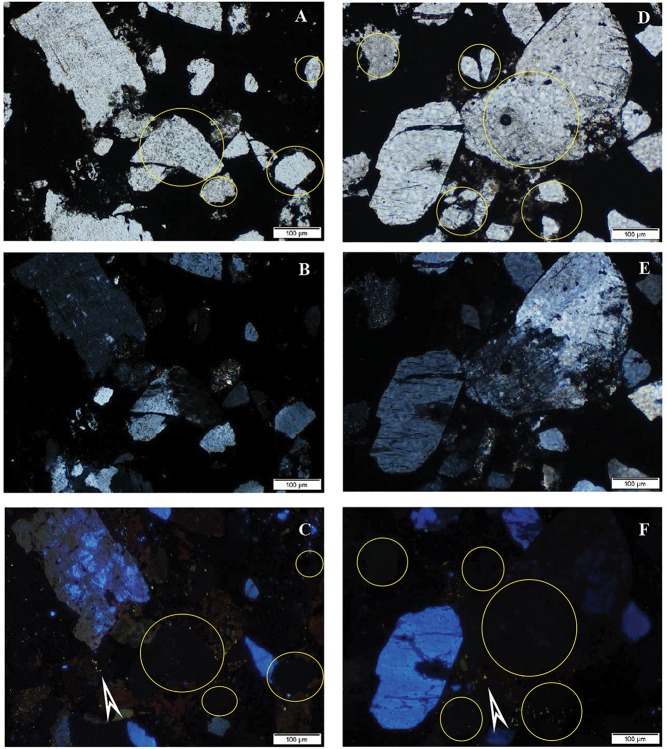
Typical cathodoluminescence images of sample COR-10. Bright blue luminescence is characteristic of feldspar and kaolinite group-dickite (supported by Raman spectroscopy, Götze et al., 2002), bright yellow minerals are small apatite grains (arrows), transmitted light photos by 1N **(A,D)**, crossed N **(B,E)**, and CL images of the same area of sample **(C,F)**. Circles show clastic-like but non-luminescent mineral grains.

A CL study revealed that a number of the debris-like grains (clastic components) are probably real clasts showing the bright, characteristic CL of the mineral (e.g., quartz – blue, feldspar – yellowish) (Figure 7). Some other grains with sizes of some tens of micrometers resembling clasts do not show luminescence. These non-luminescent grains are most probably secondary minerals, formed via diagenesis. This fact indicates authigenic mineralization via diagenesis (Marshall, 1998; Hassouta et al., 1999).

Bright blue luminescence is characteristic of feldspar and kaolinite group–dickite (supported by Raman spectroscopy, Götze et al., 2002), which occurred frequently (samples 7, 10, and 31; Figures 7C,F). The numerous small or larger bright yellow minerals are apatite grains, which often have a lighter marginal part. The apatite grains occur along the ore lenses, minerals, and laminae in a woven-like fine-grained biomat matrix, which mark the borders as accompanying a series of minerals that occurred frequently (samples 7 and 10). The fine-grained rhodochrosite (mixed carbonate) shows dull reddish (orange) luminescence (samples 7 and 10).

### Micro-Raman Spectroscopy

**FIGURE 8:**
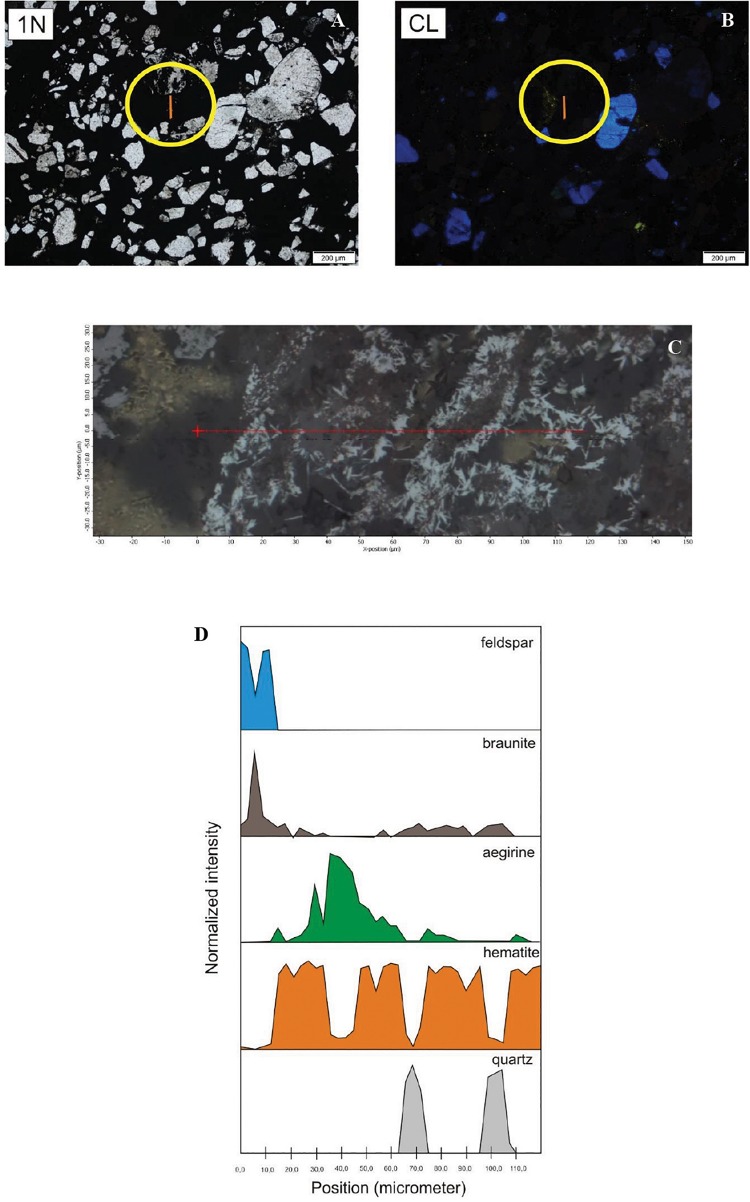
Distribution of minerals along a section in the matrix material in sample COR-10 by Raman spectroscopy (yellow line showing the section in the circle). **(A)** Transmitted light photo by 1N. **(B)** CL image. **(C)** The analyzed section marked by red line. **(D)** Mineral distribution along the section.

The 262 spectra were assessed for micromineralogical and organic matter composition as well as for the distribution of minerals according to the thin section profiles, and random point analyses. Representative analyzed profiles are shown in Figure 8. The mineral distribution was evaluated visually based on a series of Raman profiles on the 10-μm scale. The determined minerals are summarized in Table 2. Variable Mn oxides and hydroxides, Mn oxides–silicates, Mn carbonates, Fe oxides–hydroxides, Fe silicates, ore minerals, apatite, feldspar (albite and microcline), mica (muscovite), kaolinite–dickite, barite, carbonates, and quartz occur in the Mn ore beds. Variable organic material is also an important constituent. Based on low intensity and broad peaks, the minerals are poorly crystallized and cryptocrystalline. The samples contain a mixture of poorly crystallized mineral phases and organic matter. Microscale mineral phase transitions offer very important information on syngenetic and diagenetic formation processes. Mineral compositions of Urucum samples also provide information on this aspect, which explains the focus on specific mineral transitions.

**FIGURE 9:**
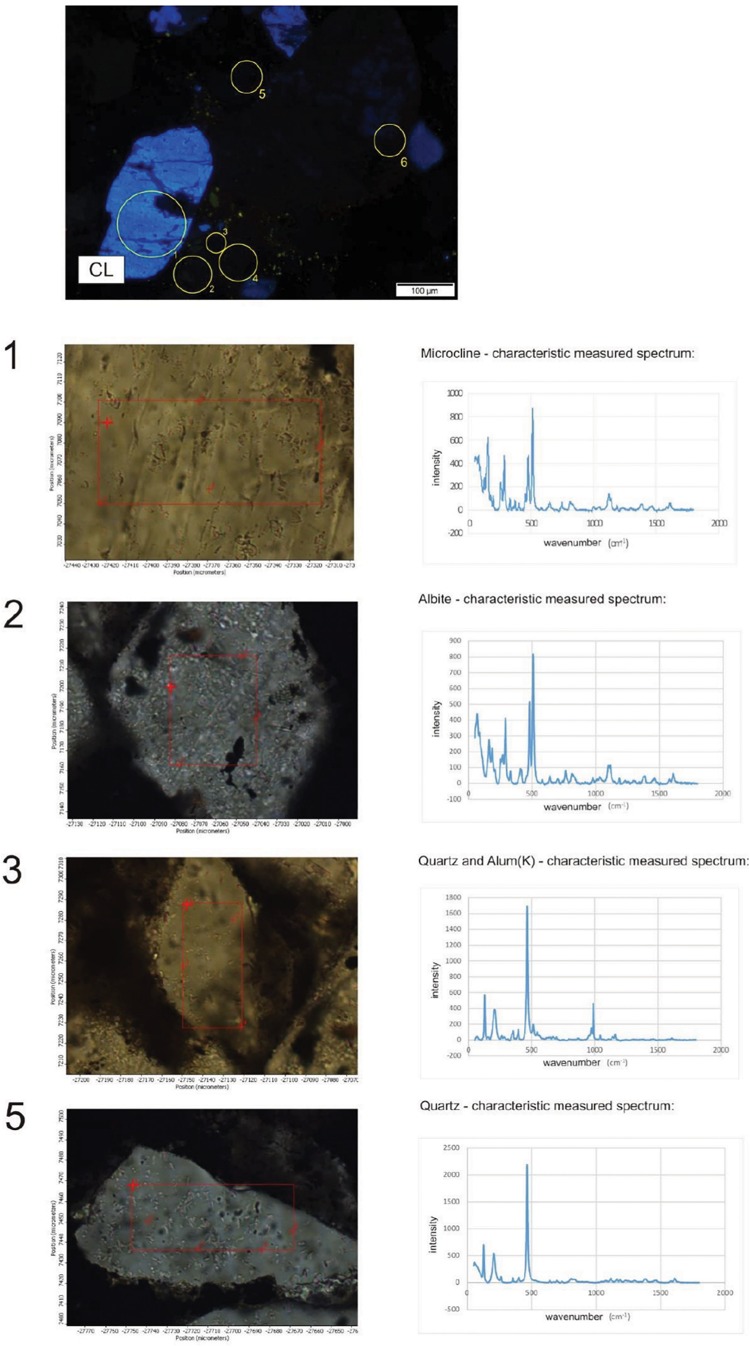
Distribution of minerals in sample COR-10. Point measurement by Raman spectroscopy. Circles and numbers on CL image show spots of analyses. Composition of grains 4 and 6 is the same as 2 (albite).

Sample COR-7 contains grains showing bright blue luminescence, which are alkali-feldspar grains based on Raman measurements. Around these luminescent grains debris-like non-luminescent grains were detected with the same mineral composition (Figure 9). The bright yellow luminescent grains are apatite.

Sample COR-10 also contains bright blue luminescent alkali-feldspars, and around them the dark brown non-luminescent grains are plagioclase. The non-luminescent quartz and feldspar grains also look like debris and often contain smaller gray, grayish-white inclusions, whose occurrence is seen to be heterogeneous by OM and obscure. The composition of these inclusions, as determined by Raman measurements, is Al-bearing sulfate, barite, and muscovite. Besides these, serandite and braunite inclusions were also detected. The bright yellow small grains are apatite here as well.

Besides random point analyses, four line maps were acquired in sample COR-10, three of which focused the grain–matrix–grain transitions (Figure 8). These line maps showed that among the non-luminescent grains, there are quartz and feldspar, which are embedded in a hematite matrix. The hematite matrix also contains barite, serandite, aegirine, and braunite. There was an apatite-bearing characteristic woven texture, along which a debris- and homogenous-like non-luminescent grain was observed. This grain showed woven, cyclic mineral laminae, and the measurements detected matrix material characterized by braunite–aegirine–hematite alternation, with scarce quartz.

Sample COR-31 contains kaolinite with a bright blue luminescent color, very similar to feldspar, which often has a rounded shape, or occurs at the margin of grains, or as irregular forms in the matrix. Dickite–hematite–organic matter composition was also detected. Marginal zones of quartz grains in some cases showed dull reddish-brown luminescence whose component is hematite, based on line map measurements.

## Discussion

### Microtextural Considerations

#### Mineralized Biosignatures

The macroscopic features suggest that the COR-7 and COR-10 samples are characterized by a very fine, granular, metallic silt. Sample COR-31 is a very fine-grained sandstone.

On the basis of OM study, the structural and morphological features indicate that all Mn-1 samples have a microlamination pattern. The microlaminae are formed by the opaque material. Well-preserved and mineralized remains of diverse filaments with pearl necklace-like, vermiform inner signatures, and coccoid-like forms embedded in the Mn ore bed were observed, and each sample is densely woven (Figure 3). This microbial microtexture is a basic feature of all of the samples, in transmitted as well as reflective light. In all thin sections, adequately high-resolution OM supports a series of mineralized biomat microstructures and mineralized microbially produced textures as the main constituents, which are similar to the Hungarian Úrkút and Chinese Datangpo deposits (Polgári et al., 2012b; Yu et al., 2019). Thus, morphological features suggest that this is a mineralized biomat, which shows the structure of the former microorganism cells. These cells probably formed an integral part of the formation environment, and their structure was retained after diagenesis. This is possible because the surface of the former cells and the EPS substance serving as the biozoenosis of the cells were mineralized during diagenesis.

#### Debris-Like Microtextural Features

Based on the cathodoluminescent microscopy analyses, it can be stated that the debris-like grains include not only fragments of clastic origin in the samples but also grains of authigenic origin, which share the same mineral quality (Figures 4–7). Some of the supposedly authigenic particles endured a transformation after debris transfer, but we have to take into account the presence of phases in the matrix material that were formed during the diagenesis. Their appearance is similar to debris at first glance, but the particles are not homogeneous, they contain a large number of inclusions, and their surface is altered and/or opaque. These grains are affected by the cell and EPS mineralization as described above.

The *in situ* Raman spectroscopy confirmed these findings. The point maps showed that the material of the samples was made mainly of feldspars (microcline, orthoclase, and albite) and quartz, and the matrix hematite contains micro-mineral phases such as apatite, barite, serandite, braunite, and aegirine. Concerning debris-like but non-luminescent mineral phases, sulfate minerals [alum(K)-KAl(SO_4_)_2_ × 12(H_2_O)] and muscovites were measured at several sites. These appear as smaller or bigger inclusions that give the grain a cloudy appearance. A similar case was detected in the Chinese Datangpo samples, where quartz grains were supposed to have a clastic origin, but their non-luminescent character supported authigenic formation (Yu et al., 2019). Gyollai et al. (2015, 2017) also reported on authigenic quartz and hydro-muscovite occurrence in the Namibian Otavi Formation of Neoproterozoic age. On the contrary, in the Hungarian Úrkút deposit, the feldspar grains show bright luminescence, confirming their tuff contribution to the sedimentary system (Polgári et al., 2012b).

#### Micro-Mineral Transformations

The line maps represent micro-mineral transformations. In these cases, we can see that the matrix material surrounding the particles is hematite, where, in some cases, 10 μm of sulfate minerals appear, such as barite or alum(K) (Table 2 and Figures 8, 9). It is assumed that their sulfate content is the result of bacterial activity. What is interesting is that the system also contains micro-sized pyroxenes that are present in the form of aegirine, supporting the results of Biondi et al. (2019). They are usually located near the quartz particles, as if they used the silicon and iron content of the quartz particles and the material of the hematite matrix in their own formation. The content of Na can be derived from the Na released from the EPS during the diagenesis. Aegirine usually occurs with a micro-sized braunite, whose Mn content can also be the result of bacterial activity. Some of the quartz in the hematite matrix is probably not debris, but perhaps gel-like segregated silica (silicon dioxide) used up by aegirine and braunite. This silica segregation is well known in case of ferrihydrite stabilization processes. According to the diagenesis of Fe-rich biomats, the microbes produce poorly ordered ferrihydrite (lepidocrocite) as a primary mineral, which transforms to more ordered minerals, such as goethite or hematite (reduced form as magnetite) within a few months or years via dissolution–dehydration processes, as mentioned before (Konhauser, 1998; Schwertmann and Cornell, 2007; Baele et al., 2008; Gyollai et al., 2015).

The Mn-1 layer in the Urucum area probably settled down in the trench structure on its first flood, settling on the fluvial, oxidative sediments of the Urucum Formation. The Mn-1 layer contains predominantly muddy and sandy, ferrous rocks that are cemented by microbially mediated Fe minerals (e.g., aegirine) and Mn oxide–silicates (braunite, serandite).

The mineralized microtextural evidence as well as the emergence of mineral species/types may indicate microbial involvement in the formation of the ore site. Presumably, two microbial ore formation systems have appeared in the formation environment, resulting in the characteristic micro-mineral associations due to the metabolic processes of the Fe- and Mn-oxidizing microorganisms.

Hematite is the matrix of the rocks of the site, in which a small amount of poorly crystallized mineral material remains (mainly in the form of quartz or feldspars). These minerals as well as microbial life activity and the decomposition of microbial EPS resulted in the varied mineralogical properties of the rock during the course of diagenesis, when a number of different minerals were formed. Due to elemental mobilization and the decomposition of complex compounds, materials deriving from microbial life activity bound in EPS caused the precipitation of aegirine instead of the formation of segregated quartz, braunite, apatite, rhodochrosite, kaolinite and smectite, the last of which appears in highly alkaline porous water in the system.

The high silica content of the system was likely to have affected the silicon dioxide uptake of the changing Mn oxide–hydroxide minerals. Through stabilization transition processes, the Mn oxide–hydroxide thereby bound silica (braunite and serandite). Braunite and serandite are Mn oxide–silicates that can also be interpreted as products of microbial activity and of the following complex diagenetic mineralization involving the usage of segregated quartz into their structure, as described in detail by Biondi et al. (2019).

Braunite formation is characterized by the diagenesis of segregated silica and hematite Fe system. The microbial influence associated with its formation is supported by the fact that the most likely sedimentary braunite formation can be realized by early diagenetic and biogeochemical processes (Serdyuchenko, 1980).

The segregated quartz produced by the microbes had a protective function in the gel matrix, which, during diagenesis, not only turned into cloudy quartz (formed at site), but also allowed the formation of braunite, serandite, and aegirine due to the Mn and Fe content of the system. Muscovite (hydromuscovite) is common in the examined samples, which was formed by the release of alkaline elements (Na, Ka, Al, Mg) of the biofilm (Ewers, 1983; Gyollai et al., 2015, 2017).

Barite and alum(K) belong to sulfates, whose formation can be linked to marine sulfates and/or life activities. EPS and its pores cause minerals to take a special forced form. The majority of minerals are cryptocrystallized.

#### Comparison—Case Studies

The Chinese study shows more similarities to the manganese ore formation found in Hungary than the Brazilian study does. There are about 480 million years between the Úrkút and the Datangpo formations; however, the processes characterizing their ore enrichment and metallogenetic mechanisms are similar (Polgári et al., 2012a, b, 2013, 2016a,b; Molnár et al., 2017; Yu et al., 2019). Both of them are Mn carbonate (Ca rhodochrosite and kutnohorite) ore in black shale environment, in which only the amount of iron differs. The Úrkút samples contain higher concentrations of iron, so we can also talk about the presence of goethite, pyrite, and celadonite in this area concerning ore minerals. The Chinese site, however, is almost free of iron and clay minerals. In terms of their formation conditions, these are ore formations taking place at low-temperature (<100°C) hydrothermal discharge zones, which were formed under oxidative conditions at the time of ore accumulation, in which later sub-oxic or locally anoxic conditions developed during the diagenesis due to embedded organic matter. Considering mineral structures, microbial traces (mineralized biostructures, mineralized biomats, and filamentous tissues, etc.) can be detected in both samples.

The Brazilian example differs from the previous ones in that it remained an oxidic ore type even after the diagenesis. Regarding the Úrkút site, three manganese cycles follow an iron cycle, while the number of iron and manganese cycles is the same for the Brazilian site, and the cycles follow each other one by one (Biondi and Lopez, 2017; Biondi et al., 2019). In the Brazilian area (unlike the other two sites), the ore-forming environment remained oxic throughout the whole process (there were no significant buffer minerals such as celadonite as in the case of Úrkút); its burying may have been slower; therefore, the majority of the organic matter was oxidized and was not buried. It also introduces a new early diagenetic ore-forming process that creates kremydilite structures. These materials, containing spherical, bubble-like spheres, also contain rhodochrosite, which may have formed in the early stages of the diagenesis by the activity of microorganisms located on organic matter during the diagenesis. This represents the second cycle in the Urucum manganese ore, which could not become a rhodochrosite-forming process forming layers due to the significantly smaller amount of organic matter, but that remained a structure around random centers. Regarding Urucum, the small amount of clay minerals was probably formed as a result of EPS transformation; therefore, there were no clay minerals with buffer effects, the presence of which would have led to rapid burying, so the burial process may have been slower than in the case of the Úrkút site.

Thus, in all three cases, the main ore formation process can be described by the two-stage microbial model; however, due to the quantity of the buried materials and their quantity ratios compared to each other, significant differences can be observed in their mineral composition.

The structural properties of iron oxide minerals allow for limited diagenetic mineral qualities (ferrihydrite, goethite, and hematite), and Fe silicates (such as aegirine) were formed together with segregating silica during the mineral stabilization.

The structure of manganese oxide and manganese hydroxide minerals allows for the widespread incorporation of cations, resulting in numerous mineral types. The cations were released from the decaying cells and the EPS, which eventually formed the manganese minerals of diverse composition. The Mn oxides also form an oxide/silicate mineral phase (serandite, braunite) with segregating silica during the process of ferrihydrite stabilization, which can be interpreted as the co-diagenesis of the two microbial cycles.

## Conclusion

Microbially mediated processes – the role of cell metabolism and EPS – are very important in syngenetic mineralization, representing also a considerable element source reservoir in the form of bond cations and anions released via diagenesis, and are very effective factors influencing the syngenetic and diagenetic mineralization processes. These processes supply bioessential elements or toxic ones, depending on the pool of elements, the type of metabolism, microbial species, and the mineralogical characteristics. In fact, they facilitate the sedimentation of metals and other elements by adsorption from the water. Economically important selective element enrichments can be mentioned, as well as the role of microbes in industrial activity and in environmental protection.

In summary, the mineralization of the cells and the EPS material contributes significantly to the material of the ore deposits, in terms of both quantity and a varied mineral composition. Microbial activity is highly effective in the mineralization and solid phase accumulation of metal ions dissolved in geofluids. It is also responsible for significant ion binding, apart from the metal enrichment processes. The organic materials of the cells and the EPS are similarly effective in the catalyzation of the processes and during the diagenetic mineralization after their degradation, which ultimately results in today’s geological properties.

The role of microorganisms is rarely considered to be significant in mineral or ore formation, as the biofilm they produce is only a millimeter thick. However, it should not be forgotten that these can cover large surfaces and thus dominate when abiotic processes occur.

## Data Availability Statement

The datasets generated for this study are available on request to the corresponding author.

## Author Contributions

MP and JB conceived and planned the study. MP and HH made macroscopic descriptions, performed the thin section microscopy, summarized and interpreted the data, and wrote the interpretation. KF made Raman measurements, interpreted the mineralogy, and made Raman profiles and CL study. IG performed the thin section microscopy and FTIR measurements, and contributed to the interpretation of data. EP-M contributed to the interpretation of data.

## Conflict of Interest

The authors declare that the research was conducted in the absence of any commercial or financial relationships that could be construed as a potential conflict of interest.

## Footnotes

1 http://rruff.info/

## Abbreviations

EPS: extracellular polymeric substance.
